# Evaluation of biomass quality in short-rotation bamboo (*Phyllostachys pubescens*) for bioenergy products

**DOI:** 10.1186/s13068-017-0818-9

**Published:** 2017-05-15

**Authors:** Seung Gon Wi, Dae-Seok Lee, Quynh Anh Nguyen, Hyeun-Jong Bae

**Affiliations:** 1 Bio-Energy Research Center, Chonnam National University, Gwangju, 500-757 Republic of Korea; 2 Department of Bioenergy Science and Technology, Chonnam National University, Gwangju, 500-757 Republic of Korea

**Keywords:** Bamboo, Bioethanol, Biomass, Cell wall composition, Short-rotation biomass

## Abstract

**Background:**

In order to improve the availability of biomass, the concept of growing high yield biomass with short rotations and intensive culture has been introduced. Bamboo has become a feedstock of potential interest for future energy production due to its high productivity and short rotation time. The growth age of biomass is an important factor affecting the efficiency of bioconversion and pretreatment for bioenergy production. In this regard, more information is required on the morphology and chemical composition of bamboo for short-rotation biomass production. In this study, we used a compositional assay to compare a bamboo of two different growth ages.

**Results:**

Bamboo of two different ages showed characteristics patterns of morphology, chemical composition, and bioconversion. In young-age (2-month-old) bamboo, the pattern of tissue organization was similar to that of old-age (3-year-old) bamboo, indicating that the former had reached its full height. There were significant differences between young-age and old-age bamboo in terms of chemical composition. The glucose contents in old-age bamboo did not differ significantly among its internodes. For young-age bamboo, the lignin contents were 14.6–18.3%, whereas those of old-age bamboo were considerably higher, ranging from 25.4 to 27.1% with increasing syringyl-to-guaiacyl ratio. The yield of total sugars following enzymatic hydrolysis of young-age bamboo was approximately eight times. However, following hydrogen peroxide–acetic acid pretreatment, the results of separate hydrolysis and fermentation and simultaneous saccharification and fermentation did not differ significantly between young- and old-age bamboo. However, ethanol production was higher in 2-month old than in 3-year old from initial raw biomass.

**Conclusion:**

Our data show that the production of total sugar from raw material was high in young bamboo with low lignin content. With respect to short-rotation biomass, bamboo culm harvested after termination of height growth is more appropriate for use as a biomass resource to achieve a high yield for bioconversion process.

## Background

As a consequence of increased energy demand and global warming, sustainability and green growth are considered key concepts for public and industrial growth. In 2014, Korea consumed 0.27 billion tons of oil equivalent (TOE) and is the 9th highest energy consumer and 7th highest carbon dioxide emitter in the world [1, 2]. Fossil fuel-based energy resources, such as petroleum (37.1%), coal (29.9%), and natural gas (16.9%), account for approximately 84% of the primary energy consumption [1]. In contrast, in 2014, the production of bioenergy as sustainable alternatives to fossil fuel-based energy resources was represented approximately by 2.8 million TOE, which was only approximately 1% of primary energy consumption [3]. Korea has limited biomass resources and the high cost of biofuels is a major barrier to their widespread use [1]. Therefore, increasing efforts are being made to identify new suitable biomass resources for biofuels production [4–8].

In order to improve the availability of biomass, the concept of growing high yield biomass with short rotations and intensive culture has been introduced [9, 10]. Bamboo has become a feedstock of potential interest for future energy production due to its high productivity, short-rotation and high economic value, and advantage for sustainable management [11, 12]. Bamboo has been used in approximately 1500 commercial application, including house, panel or composites, mat, chopsticks, sticker, charcoal or active carbon, and pulp and paper making [13]. Bamboo is a grass biomass with the hollow internode and the scattered vascular bundles throughout the stem. It belongs to the family Gramineae, which includes over 75 genera and 1250 species [13]. Worldwide, the area planted with bamboo and its annual production are estimated to be 220,000 km^2^ and 15–20 million tons, respectively [14]. Bamboo, a superior species for carbon storage, requires only 5 years to grow from shoots to mature culms [15]. In general, the development of bamboos can be classified into two growth periods [16–18]. The first stage is culm height growth with fast growth and high biomass accumulation within few months. After their full height growth, the second stage starts culms, increasing in strength and accumulating dry mass until they are mature.

It is well known that the diversity in physical and chemical properties of biomass present difficulties and challenges encountered during the processes for fuel or energy use [12]. For efficiently bioconversion of bamboo, various pretreatment methods have been used [19, 20]; however, none of these methods are cost-effective for large-scale applications. The development of a highly efficient and environmentally friendly process for the hydrolysis of cellulose into reducing sugars could be one of the key technologies for large-scale use of cellulosic biomass. In order to attain this goal, hydrogen peroxide–acetic acid (HPAC) pretreatment has been proposed and conveniently established using a mixture of green chemicals; acetic acid and hydrogen peroxide [21]. In this study, we used the HPAC pretreatment method for processing bamboo.

Growth age is an important factor affecting the efficiency of bamboo pretreatment [19]. In this regard, more information is required on the morphology and chemical composition of bamboo for short-rotation biomass production. In this study, we used a compositional assay to compare a bamboo of two different growth ages. We found a correlation between two groups for chemical composition. These compositional parameters influenced the yield obtained following pretreatment and enzymatic hydrolysis for bioenergy production.

## Results and discussion

### Biomass productivity

**Fig. 1:**
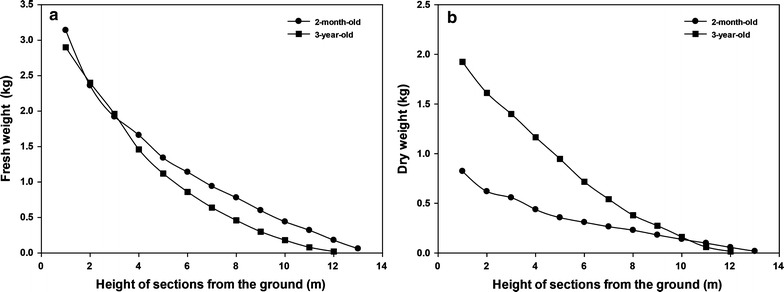
Relationships between fresh (**a**) and dry (**b**) weight and against height of each section from the ground (see “Methods” for detail). Each value is expressed as the average of three independent experiments

The productivity of bamboo was assessed on the basis of the fresh and dry weight. The biomass weight at various heights is presented in Fig. 1. The fresh and dry weight results for 2-month-old and 3-year-old bamboo showed the same trend, a decrease from the basal section (1 m) to the top section (12 or 13 m), and a decrease of biomass weight in the top section due to smaller diameter and thinner wall. The total dry weight of 3-year-old bamboo (8.43 kg/stem) was two-fold higher than 2-month-old bamboo (4.08 kg/stem); however, there was only a slight difference in total fresh weight between 3-year-old (12.38 kg/stem) and 2-month-old (14.88 kg/stem) bamboo. The moisture content in the 2-month-old and 3-year-old bamboo was 69–74 and 27–34%, respectively.

### Anatomical characteristics

**Fig. 2:**
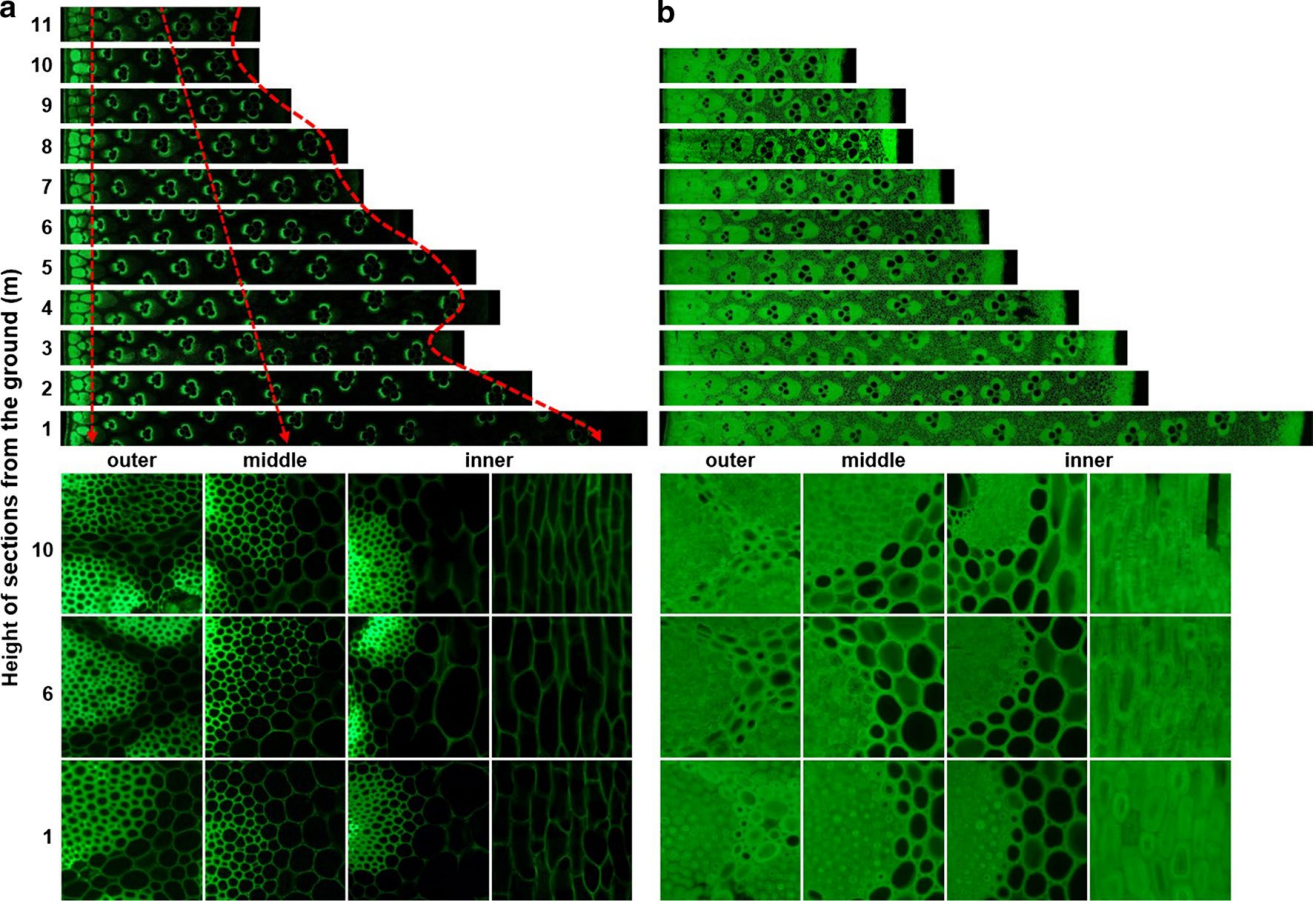
Fluorescence microscopy images of a cross-section through the section height of 2-month-old (**a**) and 3-year-old (**b**) bamboo

**Fig. 3:**
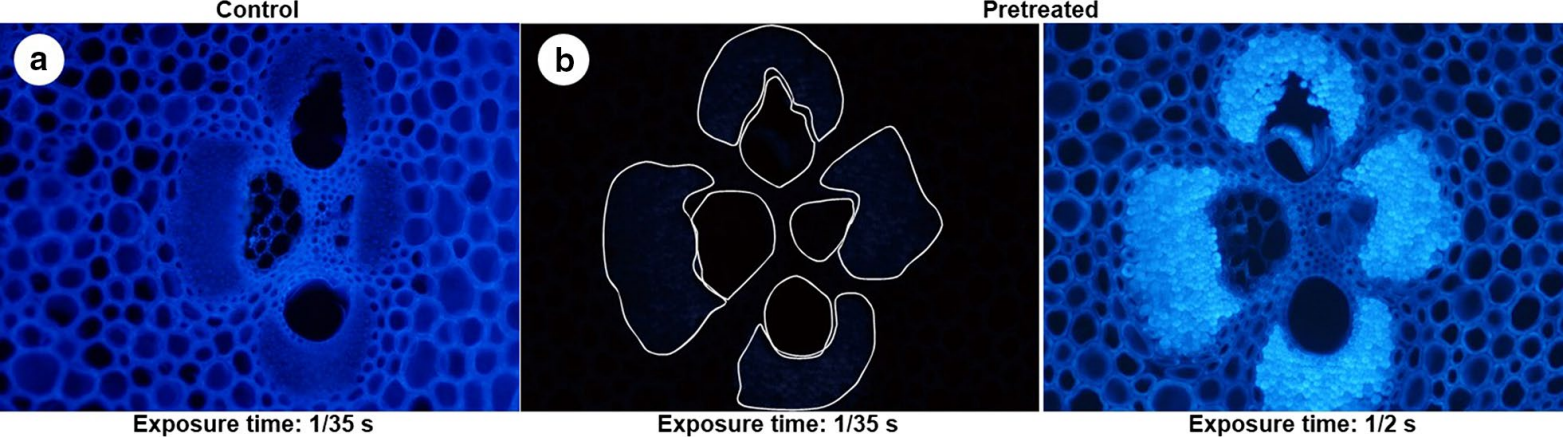
Fluorescence microscopy images of non-pretreated and hydrogen peroxide–acetic acid (HPAC) pretreated bamboo. All tissues showing autofluorescence after UV irradiation (**a**). Autofluorescence lost in all tissues after HPAC pretreatment (**b**)

As anatomical features directly affect bamboo’s physical and mechanical properties, we have conducted a comparative analysis of the anatomical characteristic between 2-month-old and 3-year-old bamboo. Autofluorescence was used to examine the morphology and lignin pattern in bamboo materials. Figure 2 shows a cross-section of bamboo stem. The internode of a bamboo stem was mainly consisted of epidermis, cortex, parenchyma, and vascular bundles. The anatomical aspects of 2-month-old bamboo were mostly similar to those of the 3-year-old bamboo; however, the cell walls of parenchyma, vascular bundle, and sclerenchyma were considerably more thickened. Fiber wall thickness did not differ significantly at different stem heights; however, there was an increase in thickness with the increase of age. Vascular bundles were surrounded by the bundle sheaths, which are distributed densely in the outer region and sparsely in the inner region. In both 2-month-old and 3-year-old bamboo, the lowest mean density of vascular bundles was in the low regions of the stem. However, the frequency of vascular bundles did not differ significantly with age. Lignin distribution was determined by using fluorescence microscopy (Fig. 3). All the tissues showed blue fluorescence after UV irradiation (Fig. 3a), but lost their autofluorescence after HPAC pretreatment (Fig. 3b). At longer exposure times (1/2 s), weak autofluorescence was detected in the middle lamella of parenchyma cells and bundle sheaths. The variation in anatomical characteristics along the bamboo column height and age presented in this study was similar to that of Huang et al. [22].

### Chemical composition

**Fig. 4:**
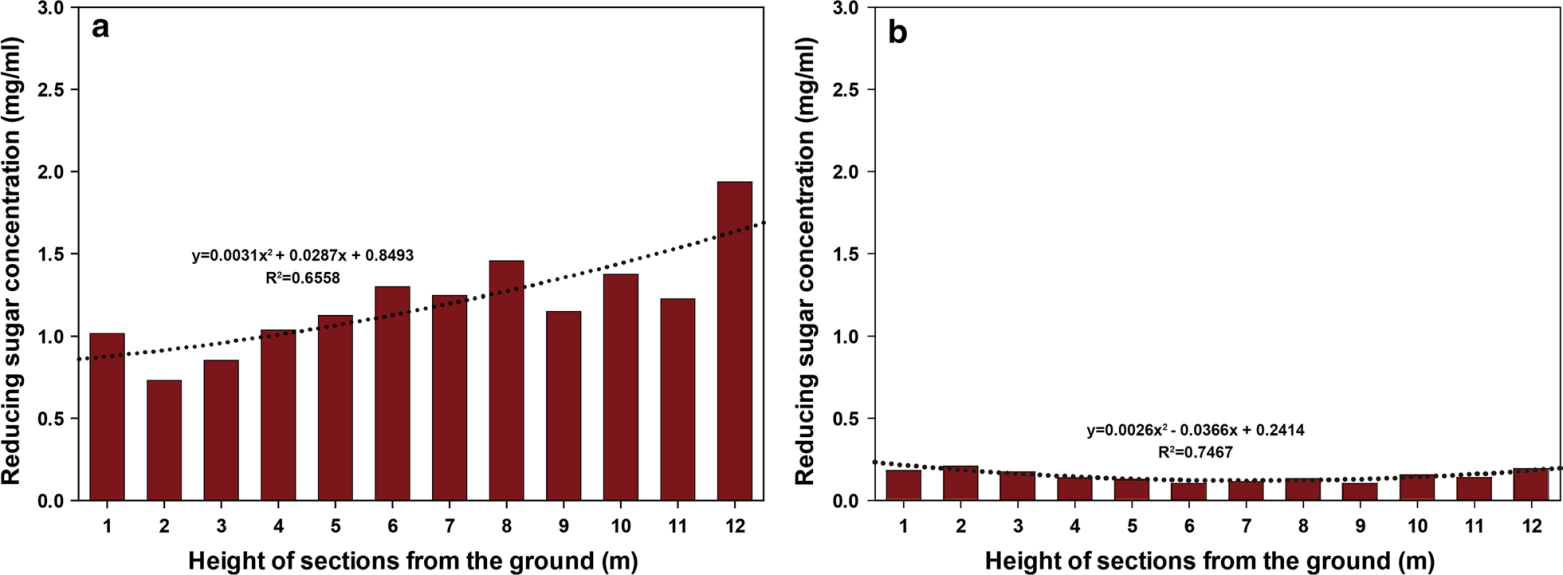
Changes with stem height in the concentrations of reducing sugar with stem height in 2-month-old (**a**) and 3-year-old (**b**) bamboo. Each value is expressed as the average of three independent experiment

The chemical composition of biomass is highly important in terms of bio-based application and variation in the relative proportions of different chemicals can influence bioenergy yield [23]. Furthermore, the chemical composition of biomass varies according to developmental stage [8, 24]. We analyzed samples collected from 2-month-old to 3-year-old bamboo stems at three different heights above ground level (1, 6, and 11 m) based on enzymatic hydrolysis data (Fig. 4), compared of the composition of bamboo cell wall (Table 1). There were significant differences between 2-month-old and 3-year-old bamboo in the contents of ash, organic solvent extractives, carbohydrate, and lignin. The contents of ash and carbohydrate in 2-month-old bamboo were higher than those in 3-year-old bamboo. However, the contents of organic solvent extractives and lignin were higher in 3-year-old bamboo. The carbohydrate contents of 2-month-old bamboo were 78.7–82.2%, whereas those of 3-year-old bamboo was 69.9–71.9%. The results of monomeric sugar and lignin analyses are shown in Table 2. Glucose and xylose were identified as major carbohydrates in the bamboo. For 2-month-old bamboo, the glucose content was highest in the 1st internode and lowest in the 11th. However, glucose contents in the different internodes of 3-year-old bamboo did not differ significantly. For 2-month-old bamboo, the lignin contents were 14.6–18.3%; however, the contents were considerably higher in 3-year-old bamboo, ranging from 25.4 to 27.1%. Furthermore, the syringyl-to-guaiacyl (S/G) ratio was higher in 3-year-old bamboo (1.25–1.45) than in 2-month-old bamboo (1.05–1.18). An increase of S/G ratio with plant maturity has been reported by Rencoret et al. [25]. The lignin content and lignin S/G ratio negatively related with enzymatic digestibility. Difference in biomass composition has a direct impact on bioenergy potential [26, 27]. In this regard, lignin [28, 29] and hemicelluloses [30] in herbaceous biomass have been shown to be negatively correlated with overall bioenergy yields. It is suggested that carbohydrate and lignin contents in bamboo, particularly at a young age, make it a useful source of biomass for bioenergy and other bio-based applications. The contents of monomeric sugars and lignin of pretreated bamboo are presented in Table 3. We focused on the three major components: glucose, xylose, and lignin. The total biomass weight of the pretreated bamboo decreased by 60–74%. While the glucose remained intact, it relatively increased in residual biomass of 2-month-old (49.4 to 62.1%) and 3-year-old bamboo (41.9 to 54.9%) due to the removal of xylose and lignin. The carbohydrates of biomass are well preserved in the HPAC process, which is a very important criterion for a pretreatment process.

**Table 1:** Compositional analysis of 2-month-old and 3-year-old bamboo

Total biomass (%)	Position height from ground (m)	Ash (%)	OSE (%)	Carbohydrate (%)	Lignin (%)		
					AIL	ASL	Total
2-month old	01	3.15 ± 0.06^a^	1.65 ± 0.09^a^	82.17 ± 0.59^c^	15.83 ± 0.89^b^	2.46 ± 0.02^a^	18.29 ± 0.89^b^
	06	4.01 ± 0.11^b^	1.75 ± 0.18^a^	80.74 ± 0.41^b^	12.31 ± 0.73^a^	2.99 ± 0.10^b^	15.30 ± 0.63^a^
	11	5.39 ± 0.13^c^	2.46 ± 0.18^b^	78.67 ± 0.26^a^	11.03 ± 0.63^a^	3.60 ± 0.10^c^	14.63 ± 0.57^a^
3-year old	01	1.36 ± 0.03^b^	5.54 ± 0.28^a^	71.85 ± 2.36	23.55 ± 0.79	1.87 ± 0.08	25.41 ± 0.84
	06	1.13 ± 0.10^a^	6.85 ± 0.43^b^	71.37 ± 2.45	23.43 ± 1.01	2.03 ± 0.11	25.46 ± 1.06
	11	1.15 ± 0.06^a^	4.75 ± 0.16^a^	69.89 ± 2.63	24.99 ± 0.72	2.13 ± 0.16	27.13 ± 0.76

If the ANOVA showed a significant difference among sample means, Scheffe’s multiple comparison test was used to determine differences. Values indicated by the same lower case letter are not significantly different (*α* = 0.01). Scheffe: a < b < c

*AIL* acid-insoluble lignin, *ASL* acid-soluble lignin, *OSE* organic solvent extractives

**Table 2:** The content of monomeric carbohydrate and lignin from extractive-free biomass of 2-month-old and 3-year-old bamboo

Extractive-free samples	Height from ground (m) position	Carbohydrate (g/kg)							Mono-lignin (g/kg)							S/G ratio
		Rham	Ara	Xyl	Man	Gal	Glu	Total	H	HA	V	VA	S	SA	Total	
2-month old	01	5.15 ± 3.56	16.95 ± 2.06	296.83 ± 8.93	5.42 ± 1.43	9.30 ± 2.81	502.15 ± 6.83^b^	835.80 ± 5.98^c^	12.73 ± 0.73^b^	0.25 ± 0.03^b^	16.46 ± 0.20^c^	0.74 ± 0.03^b^	16.70 ± 0.50^c^	3.66 ± 0.19^b^	50.55 ± 0.83^a^	1.18 ± 0.04
	06	5.02 ± 2.87	19.27 ± 4.69	297.62 ± 11.98	4.85 ± 3.08	5.80 ± 2.79	489.28 ± 7.95^b^	821.85 ± 4.20^b^	11.98 ± 0.02^b^	0.13 ± 0.02^a^	14.36 ± 0.07^b^	0.34 ± 0.02^a^	14.91 ± 0.82^b^	2.11 ± 0.56^a^	43.83 ± 0.45^b^	1.16 ± 0.07
	11	3.25 ± 0.31	18.01 ± 1.38	309.30 ± 11.19	7.21 ± 2.78	7.48 ± 1.61	461.27 ± 4.91^a^	806.53 ± 2.68^a^	8.75 ± 0.16^a^	0.28 ± 0.02^b^	10.88 ± 0.12^a^	0.40 ± 0.03^a^	9.28 ± 0.34^a^	2.53 ± 0.19^a^	32.12 ± 0.05^c^	1.05 ± 0.03
3-year old	01	2.51 ± 0.28	14.64 ± 3.06	289.49 ± 18.01	7.55 ± 3.82	2.58 ± 1.68	443.80 ± 0.76	760.61 ± 24.94	12.18 ± 1.02^b^	0.40 ± 0.05^b^	31.85 ± 1.01	0.55 ± 0.10^a^	40.80 ± 0.18^b^	3.08 ± 0.22	88.85 ± 1.78^b^	1.36 ± 0.04^a, b^
	06	2.26 ± 0.54	15.82 ± 0.35	308.80 ± 15.54	3.40 ± 2.13	4.37 ± 1.72	431.58 ± 13.89	766.21 ± 26.27	12.17 ± 0.32^b^	0.21 ± 0.04^a^	31.79 ± 1.05	0.89 ± 0.04^b^	44.17 ± 1.41^c^	3.21 ± 0.76	92.44 ± 0.60^c^	1.45 ± 0.06^b^
	11	1.75 ± 0.58	15.96 ± 0.64	302.41 ± 16.55	2.14 ± 0.91	4.10 ± 2.11	407.40 ± 12.67	733.74 ± 27.65	9.50 ± 0.17^a^	0.26 ± 0.01^a^	31.59 ± 0.63	0.95 ± 0.05^b^	37.58 ± 1.09^a^	3.13 ± 0.14	83.01 ± 0.40^a^	1.25 ± 0.05^a^

If the ANOVA showed a significant difference among sample means, Scheffe’s multiple comparison test was used to determine differences. Values indicated by the same lower case letter are not significantly different (*α* = 0.01). Scheffe: a < b < c

*Gal* galactose, *Glu* glucose, *Man* mannose, *Rham* rhamnose, *Xyl* xylose, *H* 4-hydroxybenzaldehyde, *HA* 4-hydroxybenzoic acid, *S* syringaldehyde, *SA* syringic acid, *V* vanillin, *VA* vanillic acid

**Table 3:** Effect of hydrogen peroxide–acetic acid pretreatment on 2-month-old and 3-year-old bamboo

	Pretreatment time (h)	Weight remain (%)	Carbohydrate (%)							Lignin (%)		
			Rham	Ara	Xyl	Man	Gal	Glu	Total	AIL	ASL	Total
2-month old	0.0	100.0	0.51 ± 0.12	1.67 ± 0.20^b^	29.19 ± 0.88^b^	0.53 ± 0.14^b^	0.88 ± 0.25^b^	49.39 ± 0.67^b^	82.16 ± 0.92^d^	15.91 ± 0.88^d^	2.47 ± 0.01	18.39 ± 0.87^d^
	0.5	87.3	0.18 ± 0.04	1.68 ± 0.02^b^	25.05 ± 1.39^b^	0.74 ± 0.11^b^	0.17 ± 0.06^a^	45.76 ± 0.90^a^	73.55 ± 1.82^b, c^	9.48 ± 0.40^c^	2.26 ± 0.24	11.74 ± 0.22^c^
	1.0	79.9	0.48 ± 0.04	1.59 ± 0.10^b^	25.97 ± 2.04^a, b^	0.16 ± 0.04^a^	0.24 ± 0.09^a^	45.94 ± 1.11^a^	74.38 ± 2.93^c^	2.80 ± 0.35^b^	2.55 ± 0.23	5.36 ± 0.54^b^
	1.5	75.3	0.47 ± 0.07	1.36 ± 0.20^a, b^	19.95 ± 1.24^a^	0.23 ± 0.05^a^	0.23 ± 0.05^a^	46.76 ± 0.85^a^	69.67 ± 1.11^a, b^	1.51 ± 0.26^a^	2.18 ± 0.04	3.69 ± 0.30^a^
	2.0	74.1	0.23 ± 0.05	1.20 ± 0.09^a^	19.73 ± 1.82^a^	0.16 ± 0.04^a^	0.32 ± 0.03^a^	46.19 ± 1.21^a^	67.84 ± 0.72^a^	1.12 ± 0.01^a^	2.64 ± 0.39	3.76 ± 0.39^a^
3-year old	0.0	100.0	0.24 ± 0.03	1.38 ± 0.29^b^	27.35 ± 1.70^c^	0.71 ± 0.36^b^	0.24 ± 0.16	41.92 ± 0.76^b^	71.85 ± 2.36^c^	23.99 ± 0.19^c^	1.90 ± 0.05^a^	25.89 ± 0.14^d^
	0.5	80.6	0.31 ± 0.11	1.13 ± 0.07^a, b^	21.28 ± 1.75^b^	0.32 ± 0.02^a, b^	0.24 ± 0.04	36.51 ± 1.37^a, b^	59.80 ± 2.85^b^	9.91 ± 1.13^b^	2.42 ± 0.14^b^	12.33 ± 1.01^c^
	1.0	67.6	0.21 ± 0.06	0.87 ± 0.06^a^	17.50 ± 1.30^a^	0.14 ± 0.03^a^	0.14 ± 0.05	35.63 ± 2.49^a, b^	54.49 ± 2.46^a, b^	1.56 ± 0.11^a^	2.23 ± 0.33^a, b^	3.78 ± 0.27^b^
	1.5	60.7	0.30 ± 0.11	0.79 ± 0.08^a^	16.76 ± 0.32^a^	0.06 ± 0.01^a^	0.07 ± 0.01	38.19 ± 3.73^a, b^	56.16 ± 3.54^a, b^	0.54 ± 0.08^a^	1.94 ± 0.16^a, b^	2.49 ± 0.19^a^
	2.0	60.0	0.24 ± 0.01	0.78 ± 0.06^a^	15.96 ± 0.93^a^	0.27 ± 0.02^a^	0.18 ± 0.02	32.94 ± 2.04^a^	50.36 ± 2.34^a^	0.30 ± 0.07^a^	1.84 ± 0.06^a^	2.14 ± 0.04^a^

If the ANOVA showed a significant difference among sample means, Scheffe’s multiple comparison test was used to determine treatment difference. Values indicated by the same lower case letter are not significantly different (*α* = 0.01). Scheffe: a < b < c < d

*AIL* acid-insoluble lignin, *ASL* acid-soluble lignin, *Gal* galactose, *Glu* glucose, *Man* mannose, *Rham* rhamnose, *Xyl* xylose

### Effects of stem height position on enzymatic hydrolysis

Bamboo stems were divided into 1-m sections, and the relationship between their chemical composition and enzymatic hydrolysis yield was compared (Fig. 4). The saccharification yield was approximately eight times higher in 2-month-old bamboo than in 3-year-old bamboo. Reducing sugar concentration in the hydrolysate of 2-month-old bamboo was 0.7–1.9 mg/mL, whereas that of 3-year-old bamboo was 0.1–0.2 mg/mL. The saccharification pattern on each internode differed greatly between bamboos of the two different ages. Saccharification was the highest in the upper stem and the lowest in the lower position of 2-month-old bamboo, whereas in 3-year-old bamboo, it was higher in the upper and lower parts of the stem and lower in middle-stem region. Accordingly, at the same sample height, the saccharification yield was inversely related to the S/G ratio on sample position (Table 2).

### Separate hydrolysis and fermentation (SHF) and simultaneous saccharification and fermentation (SSF)

**Fig. 5:**
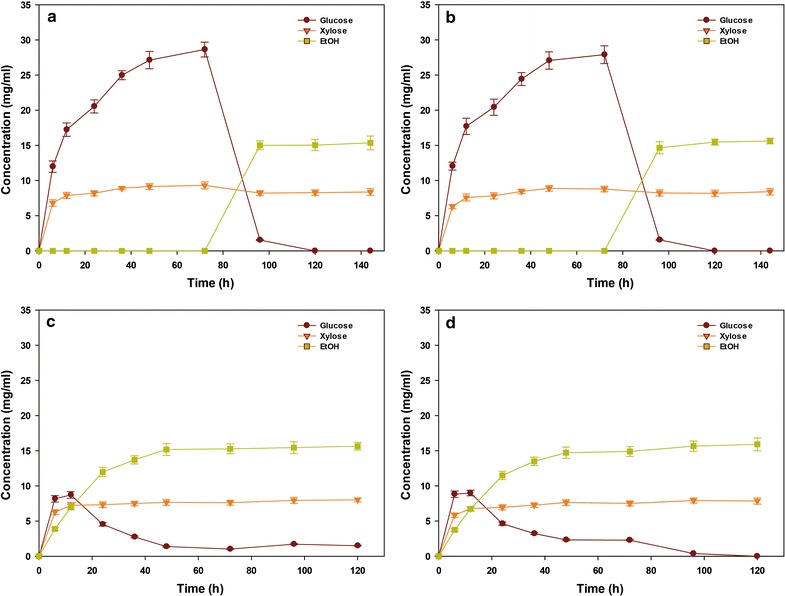
Concentration profiles for SHF (**a**, **b**) and SSF (**c**, **d**) process of HPAC-pretreated 2-month-old (**a**, **c**) and 3-year-old (**b**, **d**) bamboo at 5% solid loading

**Fig. 6:**
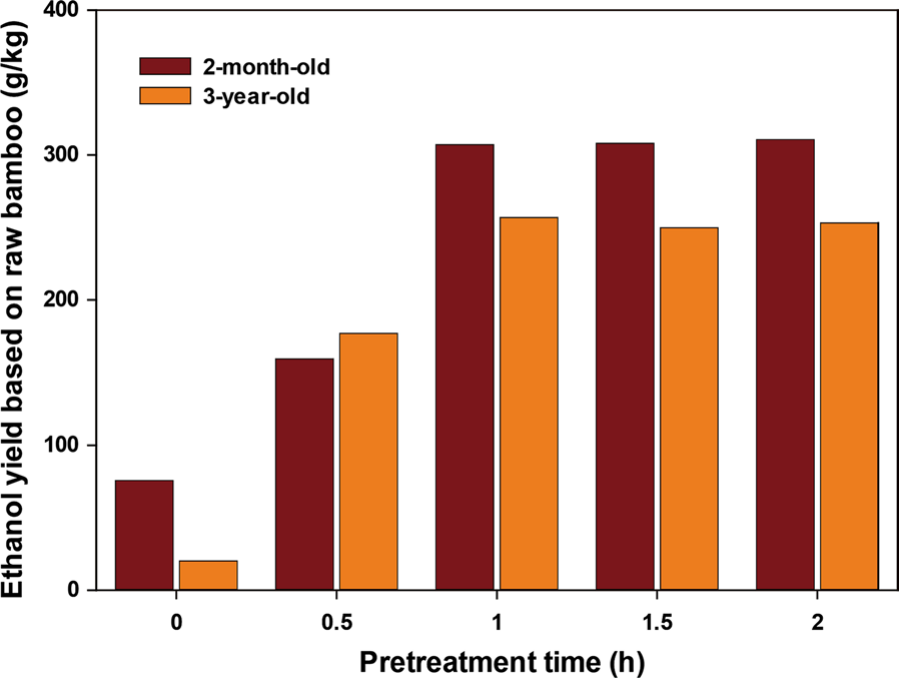
Effect of pretreatment time on ethanol productivity based on the raw bamboo. Each value is expressed as the average of three independent experiments

SHF and SSF processes were performed at 5% (w/v) solid loading (Fig. 5). In SHF process, enzymatic hydrolysis of HPAC-pretreated 2-month-old bamboo resulted in a 28.6 g/L glucose during 72 h; corresponding to an overall glucose recovery of 91.8% (Fig. 5a). In the case of 3-year-old bamboo, the final concentration and recovery of glucose were 27.9 g/L and 88.6%, respectively (Fig. 5b). After supplementation with yeast, the ethanol concentration in 2-month-old and 3-year-old bamboo reached 15.3 and 15.6 g/L, respectively, which was based on total glucose content in HPAC-pretreated biomass assuming 96.9 and 96.1% fermentation yield within a 72-h period (Fig. 5a, b). Based on the theoretical yield of 0.51 g ethanol/g glucose, the final ethanol concentration after fermentation was higher than the amount of enzymatically released glucose in hydrolysates. The reason may be cellobiose which was hydrolyzed into glucose by β-glucosidase during the fermentation period. This assumption is in agreement with the previous report that enzymatic saccharification is not only a function of the raw biomass and enzyme cocktail, but it is also dependent on the required residence time [31]. In the SSF process, the SSF pattern in the bamboo of different ages was similar. The ethanol concentration in 2-month-old and 3-year-old bamboo after 72-h fermentation was 15.2 and 14.9 g/L, respectively, which was 96 and 93% of the theoretical yield based on hexose conversion (Fig. 5c, d). However, ethanol fermentation of HPAC-pretreated bamboo achieved 307 g (2-month-old) and 257 g (3-year-old) of ethanol of 1 kg of initial raw biomass (Fig. 6). These results demonstrate that the biological conversion of young bamboo to ethanol is efficient with pretreatment.

## Conclusions

Bamboo is considered a grass biomass material that has great potential as a future bioresource for biorefining. The bamboo of two different ages examined in the present study showed characteristics pattern in terms of morphology, chemical composition, and bioconversion. Two-month-old bamboo had a pattern of tissue organization similar to that of 3-year-old bamboo, indicating that it had reached its full height. There were significant differences in the chemical compositions of 2-month-old and 3-year-old bamboo. The glucose contents in 3-year-old bamboo did not differ significantly among its internodes. For 2-month-old bamboo, the lignin contents were 14.6–18.3%, whereas those in 3-year-old bamboo were considerably higher, ranging from 25.4 to 27.1%. Furthermore, S/G ratio was higher in 3-year-old bamboo compared to 2-month-old bamboo. Total sugar yield from enzymatic hydrolysis was approximately eight times higher in 2-month-old than in 3-year-old raw bamboo. The results of SHF and SSF for 2-month-old and 3-year-old bamboo after HPAC pretreatment were not significantly different. However, ethanol yield after HPAC pretreatment was higher in 2-month old than in 3-year old from initial raw biomass. Our data showed that the productivity of total sugar from raw material was high in 2-month-old bamboo with low lignin content. In terms of short-rotation biomass production, bamboo culms harvested after termination of height growth are suitable for use as a biomass resource to achieve a high yield for bioconversion process.

## Methods

### Biomass and pretreatment

In this study, we used bamboos (*Phyllostachys pubescens*) of age 2 months and 3 years, grown at the campus of Chonnam National University in Gwangju, South Korea. Columns were harvested at the end of May 2016. The columns were subsequently subdivided into 1 m length, labeled 1(basal) to 13(top), and then freeze-dried. These bamboo samples were ground in a Willy Mill preferably to a particle size sufficiently small to pass through a 40-mesh to 60-mesh sieve.

Ground bamboo samples (positioned 1, 6, and 11 m above ground) were pretreated using HPAC method [21]. Briefly, the pretreatment solution was prepared by mixing hydrogen peroxide and acetic acid (1:1; v/v). Ten grams of each sample were treated separately with 50 mL of the reagent and kept at 80 °C for 2 h. Pretreated samples for the chemical analysis and enzymatic saccharification were collected at 30-min intervals, washed extensively with distilled water until a neutral pH was attained, and then freeze-dried for 48 h.

### Fluorescence microscopy

Sections (10–20 μm thick) were cut transversely through bamboo stem at each height. Sections without staining were mounted in water and observed through autofluorescence. To compare the morphological and histochemical differences before and after pretreatment, cross-sections were pretreated using the above-mentioned condition and then gently washed with distilled water. Autofluorescence was recorded using a UV filter cube (excitation filter band pass 330 to 385 nm and barrier filter 420 nm) [32].

### Chemical composition

The chemical composition [Klason lignin (T 222 om-88), organic solvent extractives (T 204 om-88), and ash (T 211 om-85)] of raw and pretreated bamboo stem was analyzed in accordance with the TAPPI Standard Method [33].

### Structural carbohydrates

Structural carbohydrate of samples was analyzed using gas chromatography [6, 34]. Each sample was treated with sulfuric acid for 1 h at room temperature, and hydrolysis was performed at 121 °C for 1 h. Myo-inositol was added to the samples as an internal standard, and the mixture was neutralized with ammonia water. The reactant was reduced to alditols with sodium tetrahydroborate, and the excess sodium tetrahydroborate was decomposed with acetic acid. Alditols were acetylated with acetic anhydride through methylimidazole catalysis and then extracted with dichloromethane. This solution was analyzed using a gas chromatograph (GC-2010; Shimadzu, Otsu, Japan) equipped with a DB-225 capillary column (30 m × 0.25 mm ID, 0.25-μm film thickness, J & W Scientific, CA, USA) operated with helium. The operation conditions were as follows: injector temperature 220 °C; flame ionization detector (FID) temperature 250 °C; initial oven temperature 100 °C for 1.5 min; and heating rate 5 °C/min up to 220 °C [6].

### Lignin monomers

The lignin monomers were determined by alkaline nitrobenzene oxidation [35]. Thirty milligrams of sample in a 10-mL stainless steel reactor were suspended in 4 mL NaOH (2 M). To this mixture, 0.25 mL nitrobenzene was added, followed by heating at 170 °C for 2 h. The reactor was then immediately placed in ice water, followed by the addition of 0.1 mL ethyl-vanillin (50 mg/mL). After extraction with dichloromethane and ether, the solvent layer was transferred to a beaker filled with sodium sulfate to remove water, and was subsequently evaporated. The dried products were reacted with *N*,*O*-bis(trimethylsilyl)-acetamide at 105 °C for 2–3 min. The trimethylsilyl-derivatized solution was then analyzed using a gas chromatograph (CP-9100, Chrompack, The Netherlands) equipped with a CP-Sil 5CB-fused silica capillary column (25 m × 0.32 mm ID, 1.2-μm film thickness, The Chrompack, Netherlands) operated with helium. The operation conditions were as follows: injector temperature 280 °C; flame ionization detector (FID) temperature 280 °C; initial oven temperature 150 °C for 5 min; and heating rate 10 °C/min up to 250 °C [8].

### Enzyme assays and enzymatic hydrolysis

Cellulase (Celluclast 1.5 L, Novozyme) and xylanase (X2753, Sigma) were used for enzymatic hydrolysis. Cellulase and xylanase activities were measured according to Adney and Baker [36] and Teixeira et al. [37], respectively. Enzyme activities for cellulase and xylanase were 69.4 filter paper units (FPU)/mL and 979 IU/mL, respectively.

Enzymatic hydrolysis was conducted at 1% dry matter (DM, w/v) initial substrate loading in a 50-mL conical tube. A substrate was soaked in 0.05 M sodium citrate buffer (pH 5), with sodium azide (0.2%, w/v) added as an antibiotic to prevent microbial contamination. Enzymatic hydrolysis was performed at 37 °C with an enzyme loading of 10 FPU cellulase/g^1^ biomass and 1000 IU xylanase/g^1^ biomass for 72 h [7]. The sugar contents in these hydrolysates were measured using DNS assay [38] and HPLC method [7].

### Separate hydrolysis and fermentation (SHF) and simultaneous saccharification and fermentation (SSF)

Enzymatic saccharification was conducted in a 500-mL Erlenmeyer flask with a total working volume of 100 mL at a substrate concentration of 5% DM (w/v) with 0.1% (w/v) yeast extract, 0.2% (w/v) peptone, and 0.05 M citrate buffer (pH 4.8). Reaction flasks were run with an enzyme loading of 10 FPU cellulase and 1000 IU xylanase/g biomass at 150 rpm. After 72 h, *Saccharomyces cerevisiae* KCTC 7906 (50 mg of dry yeast) was added to 100 mL of hydrolysates. Fermentation was carried out at 32 °C for 72 h with agitation at 150 rpm [7]. SSF was conducted in a 100 mL total volume containing 5% DM (w/v), cellulase (10 FPU/g biomass), xylanase (1000 IU/g biomass), 50 mg dry yeast (*S*. *cerevisiae* KCTC 7906), 0.1% (w/v) yeast extract, 0.2% (w/v) peptone, and 0.05 M citrate buffer (pH 4.8) at 37 °C for 120 h in a 500-mL Erlenmeyer flask [8].

Sugars (glucose and xylose) and ethanol produced during SHF and SSF processes were monitored using an HPLC system equipped with a refractive index detector (YoungLin Instruments, Anyang, Korea). A Rezex ROA organic acid column (300  ×  7.8 mm, Phenomenex, Torrance, CA) was used for compound identification. The temperatures of the column and detector were maintained at 65 and 40 °C, respectively, and 5 mM sulfuric acid was used as the mobile phase [7]. Fermentation efficiency was calculated on the basis of total glucose content in the pretreated materials by dividing the quantity of ethanol produced by the total amount of glucose.

### Data analysis

The SPSS software was used for statistical analysis. One-way ANOVA was used to test. Additionally, the Scheffe’s test (at *α* = 0.01) was applied to detect differences in group means.

## Abbreviations

AIL: acid-insoluble lignin
ASL: acid-soluble lignin
DM: dry matter
FPU: filter paper unit
Gal: galactose
GC: gas chromatography
Glu: glucose
Man: mannose
H: 4-hydroxybenzaldehyde
HA: 4-hydroxybenzoic acid
HPLC: high-performance liquid chromatography
HPAC: hydrogen peroxide–acetic acid
IU: International unit
OSE: organic solvent extractives
Rham: rhamnose
S: syringaldehyde
SA: syringic acid
SHF: separate hydrolysis and fermentation
S/G ratio: syringyl-to-guaiacyl ratio
SSF: simultaneous saccharification and fermentation
V: vanillin
VA: vanillic acid
Xyl: xylose
